# Supplementary material for: Inner surface of Nepenthes slippery zone: ratchet effect of lunate cells causes anisotropic superhydrophobicity
Source: R Soc Open Sci. 2020 Mar 25;7(3):200066. doi: 10.1098/rsos.200066 (PMC7137952; doi:10.1098/rsos.200066)
Supplement: Derivation of Equations [file rsos200066supp1.docx]

**Electronic supplementary Material (ESM) for**

**Inner surface of *Nepenthes* slippery zone: Ratchet effect of lunate cells causes anisotropic superhydrophobicity**

Lixin Wang^1^, Shuoyan Zhang^1^, Shanshan Li^1^, Shixing Yan^2^, Shiyun Dong^2^

^1^School of Mechanical Engineering, Hebei University of Science and Technology, Shijiazhuang 050018, People’s Republic of China

^2^National Key Laboratory for Remanufacturing, Academy of Armord Forces Engineering, Beijing 100072, People’s Republic of China

Author for correspondence: Lixin Wang, e-mail: [wanglx@hebust.edu.cn](mailto:wanglx@hebust.edu.cn)

ID: LW, 0000-0002-4205-5638

**Running Head:** Ratchet effect causes the anisotropy

**S1. Derivation of the equations concerning sliding angle and structural characteristics of lunate cells**

Volume of the ultrapure-water droplet used in measuring the wettability of *Nepenthes* slippery zone is $V$=3$\mu\text{L}$, and generated the static contact angle$\theta=155.70\pm1.05^{\circ}$. Thus, the radius ($R$=0.895 mm) of ultrapure-water droplet and its contact radius ($r$=0.368 mm) on the slippery zone can be calculated.

To characterize the anisotropic wettability of slippery zone, we measured the sliding angle of an ultrapure-water droplet along downward (toward pitcher bottom) and upward (toward pitcher up) direction. At first, the slippery zone was horizontally fixed on a measuring platform and an ultrapure-water droplet was dropped to the slippery zone, and then applied a sliding angle. As shown in Fig. 4A and B, applying a sliding angle $\theta_{\text{sa}}$means raising the ultrapure-water droplet to a height, and thus obtains a gravitational potential energy $W_{\text{l}}$. The resistance, such as the gravitational potential energy $W_{\text{s}}$ and $W_{\text{p}}$ respectively produced by the slope (Fig. 4A) and precipice (Fig. 4B) of lunate cell, as well as the work done by adhesion force $W_{\text{a}}$ and friction force $W_{\text{f}}$ provided by the interaction between the ultrapure-water droplet and the slippery zone (lunate cell, wax coverings), would restrict the rolling of ultrapure-water droplet. According to the law of energy conservation, the necessary condition for the rolling is

$W_{\text{l}}\geq W_{\text{s}}\text{（}W_{\text{p}}\text{）}+W_{\text{a}}+W_{\text{f}}$. (1)

When the ultrapure-water droplet is rolling toward the pitcher bottom (Fig. 4A, C), it should overcome the restriction of the gravitational potential energy $W_{\text{s}}$ produced by the ‘slope’ structure, and the work done by the adhesion/friction force $W_{\text{a-s}}$/$W_{\text{f-s}}$ of the ultrapure-water droplet on the ‘slope’ structure and the wax coverings (Fig. 4E) among lunate cells along the horizontal direction.

For the gravitational potential energy $W_{\text{l-s}}$ of the ultrapure-water droplet induced by the sliding angle$\theta_{\text{sa-s}}$, we can deduce the following equation

$W_{\text{l-s}}\text{=}mgr\sin\theta_{\text{sa-s}}$. (2)

For the work done by adhesion force$W_{\text{a-s}}$, we can obtain the following equation

$W_{\text{a-s}}=\Delta S_{\text{s}}\gamma_{\lg}(1+\sin\theta+\cos\theta)$, (3)

where $\Delta S_{\text{s}}$ represents the wetting area when the ultrapure-water droplet rolls from underside to upside of the ‘slope’ structure. It includes the rectangular area (aa´b´b, Fig. 4C), and the triangular areas (abc and a´b´c´, Fig. 4C), as well as the wax coverings among the ‘slope’ structures (rectangular area aa´b´b, Fig. 4E) along the horizontal direction, namely perpendicular to the ultrapure-water rolling direction. $\gamma_{\lg}$ is the surface tension of ultrapure-water, and $\theta$ stands for the static contact angle of the ultrapure-water droplet on the slippery zone. We assume that when from underside to upside, the number of lunate cells wetted by the ultrapure-water droplet is $n$, thereby the following equation can be deduced,

$\begin{matrix} \Delta S_{\text{s}}=2r\frac{h_{s}}{\tan\alpha_{\text{s}}}+n(\frac{h_{s}}{\sin\alpha_{\text{s}}}L+\frac{h_{s}^{2}}{\tan\alpha_{\text{s}}})-n\frac{h_{s}}{\tan\alpha_{\text{s}}}L \\ =\frac{2rh_{s}}{\tan\alpha_{\text{s}}}+n[(\frac{h_{s}}{\sin\alpha_{\text{s}}}-\frac{h_{s}}{\tan\alpha_{\text{s}}})L+\frac{h_{s}^{2}}{\tan\alpha_{\text{s}}}] \end{matrix}$ (4)

where $h_{\text{s}}$/$\alpha_{\text{s}}$ is the height/angle of ‘slope’ structure along pitcher bottom direction, $L$ is the length of lunate cell. Therefore, the work done by the adhesion force can be exhibited by the following equation

$W_{\text{a-s}}=\gamma_{\lg}[\frac{2rh_{\text{s}}}{\tan\alpha_{\text{s}}}+n(\frac{h_{\text{s}}}{\sin\alpha_{\text{s}}}-\frac{h_{\text{s}}}{\tan\alpha_{\text{s}}})L+n\frac{h_{\text{s}}^{2}}{\tan\alpha_{\text{s}}}](1+\sin\theta+\cos\theta)$ (5)

We assume that the mass of the ultrapure-water droplet which wets the ‘slope’ structure is $m_{\text{lc}}$, and the mass of the ultrapure-water droplet which wets the wax coverings among the lunate cells (along horizontal direction) is $m_{\text{wc}}$, thus the whole mass of the ultrapure-water droplet is $m\text{=}m_{\text{lc}}+m_{\text{wc}}$. For the work done by friction force $W_{\text{f-s}}$, $W_{\text{f-s}}\text{=}\mu F_{\text{ns}}\Delta x_{\text{s}}$, where $\mu$is the friction coefficient between the ultrapure-water droplet and the slippery zone, $F_{\text{ns}}$ is the normal force provided by the ‘slope’ structure or the wax coverings to the ultrapure-water droplet, $\Delta x_{\text{s}}$ represents the displacement of the ultrapure-water droplet rolling (wetting) from underside to upside of the ‘slope’ structure.

For the region of ‘slope’ structure (Fig. 4A), the normal force $F_{\text{ns-lc}}\text{=}m_{\text{lc}}g\cos(\alpha_{\text{s}}-\theta_{\text{sa-s}})$, the displacement$\Delta x_{\text{s-lc}}\text{=}\frac{h_{s}}{\sin\alpha_{\text{s}}}$, thus we obtain the work done by the ‘slope’ structure, as flows:

$W_{\text{f-s-lc}}\text{=}\mu m_{\text{lc}}g\cos(\alpha_{\text{s}}-\theta_{\text{sa-s}})\frac{h_{s}}{\sin\alpha_{\text{s}}}$, (6)

namely,

$W_{\text{f-s-lc}}\text{=}\mu m_{\text{lc}}g\cos\theta_{\text{sa-s}}\frac{h_{s}}{\tan\alpha_{\text{s}}}+\mu m_{\text{lc}}gh_{s}\sin\theta_{\text{sa-s}}$. (7)

For the region of wax coverings among the lunate cells along the horizontal direction (Fig. 4E), the normal force $F_{\text{ns-wc}}\text{=}m_{\text{wc}}g\cos\theta_{\text{sa-s}}$, and the displacement $\Delta x_{\text{s-wc}}\text{=}\frac{h_{s}}{\tan\alpha_{\text{s}}}$, thus we obtain the work done by the wax coverings, as flows:

$W_{\text{f-s-wc}}\text{=}\mu m_{\text{wc}}g\cos\theta_{\text{sa-s}}\frac{h_{s}}{\tan\alpha_{\text{s}}}$. (8)

Therefore, the work done by the friction force $W_{\text{f-s}}$ can be shown by the following equation

$\begin{matrix} W_{\text{f-s}}\text{=}W_{\text{f-s-lc}}+W_{\text{f-s-wc}}\text{=}\mu m_{\text{lc}}g\cos\theta_{\text{sa-s}}\cos\alpha_{\text{s}}\frac{h_{s}}{\sin\alpha_{\text{s}}}+\mu m_{\text{wc}}g\cos\theta_{\text{sa-s}}\frac{h_{s}}{\tan\alpha_{\text{s}}}+\mu m_{\text{lc}}gh_{s}\sin\theta_{\text{sa-s}} \\ \text{=}\mu(m_{\text{lc}}g+m_{\text{wc}}g)\cos\theta_{\text{sa-s}}\frac{h_{s}}{\tan\alpha_{\text{s}}}+\mu m_{\text{lc}}gh_{s}\sin\theta_{\text{sa-s}} \\ \text{=}\mu mg\cos\theta_{\text{sa-s}}\frac{h_{s}}{\tan\alpha_{\text{s}}}+\mu m_{\text{lc}}gh_{s}\sin\theta_{\text{sa-s}} \end{matrix}$. (9)

Finally, according to the $W_{\text{l}}\geq W_{\text{s}}+W_{\text{a}}+W_{\text{f}}$, we can conduct the following deduction

$\begin{matrix} mgr\sin\theta_{\text{sa-s}}=mgh_{\text{s}}+\gamma_{\lg}[\frac{2rh_{\text{s}}}{\tan\alpha_{\text{s}}}+n(\frac{h_{\text{s}}}{\sin\alpha_{\text{s}}}-\frac{h_{\text{s}}}{\tan\alpha_{\text{s}}})L+n\frac{h_{\text{s}}^{2}}{\tan\alpha_{\text{s}}}](1+\sin\theta+\cos\theta)+ \\ \mu mg\cos\theta_{\text{sa-s}}\frac{h_{\text{s}}}{\tan\alpha_{\text{s}}}+\mu m_{\text{lc}}gh_{s}\sin\theta_{\text{sa-s}} \end{matrix}$, (10)

namely,

$\begin{matrix} (mr\text{-}\mu m_{\text{lc}}h_{s})g\sin\theta_{\text{sa-s}}=mgh_{\text{s}}+\gamma_{\lg}[\frac{2rh_{\text{s}}}{\tan\alpha_{\text{s}}}+n(\frac{h_{\text{s}}}{\sin\alpha_{\text{s}}}-\frac{h_{\text{s}}}{\tan\alpha_{\text{s}}})L+n\frac{h_{\text{s}}^{2}}{\tan\alpha_{\text{s}}}](1+\sin\theta+\cos\theta)+ \\ \mu mg\cos\theta_{\text{sa-s}}\frac{h_{\text{s}}}{\tan\alpha_{\text{s}}} \end{matrix}$. (11)

In the above equation, $m>m_{\text{lc}}$, $\mu=0.1$, $r=368.2$µm, $h_{\text{s}}\text{=5.39}$µm, so $mr\gg\mu m_{\text{lc}}h_{\text{p}}$, and the $\mu m_{\text{lc}}h_{\text{p}}$ can be ignored. Thereby, we can obtain the following equation

$\begin{matrix} mrg\sin\theta_{\text{sa-s}}=mgh_{\text{s}}+\gamma_{\lg}[\frac{2rh_{\text{s}}}{\tan\alpha_{\text{s}}}+n(\frac{h_{\text{s}}}{\sin\alpha_{\text{s}}}-\frac{h_{\text{s}}}{\tan\alpha_{\text{s}}})L+n\frac{h_{\text{s}}^{2}}{\tan\alpha_{\text{s}}}](1+\sin\theta+\cos\theta)+ \\ \mu mg\cos\theta_{\text{sa-s}}\frac{h_{\text{s}}}{\tan\alpha_{\text{s}}} \end{matrix}$. (12)

Based on the same method, when the ultrapure-water droplet is rolling toward the pitcher up (Fig. 4B, D and E), namely along upward direction, the following equation can be deduced,

$\begin{matrix} mrg\sin\theta_{\text{sa-p}}=mgh_{\text{p}}+\gamma_{\lg}[\frac{2rh_{\text{p}}}{\tan\alpha_{\text{p}}}+n(\frac{h_{\text{p}}}{\sin\alpha_{\text{p}}}-\frac{h_{\text{p}}}{\tan\alpha_{\text{p}}})L+n\frac{h_{\text{p}}^{2}}{\tan\alpha_{\text{p}}}](1+\sin\theta+\cos\theta)+ \\ \mu mg\cos\theta_{\text{sa-p}}\frac{h_{\text{p}}}{\tan\alpha_{\text{p}}} \end{matrix}$, (13)

where $\theta_{\text{sa-p}}$ is the sliding angle of the ultrapure-water droplet rolling on the slippery zone along pitcher up direction, $h_{\text{p}}$/$\alpha_{\text{p}}$ is the height/angle of ‘precipice’ structure along pitcher up direction.

According to the Equation (12) and Equation (13), and the structural parameters of lunate cell, the theoretical sliding angle $\theta_{\text{sa-st}}$/$\theta_{\text{sa-pt}}$ can be calculated.
